# Patient reported outcomes and results of endometrial balloon ablation for the treatment of heavy menstrual bleeding: A single center observational cohort study

**DOI:** 10.1002/ijgo.71228

**Published:** 2026-07-09

**Authors:** Jonas Rudaitis, Patricija Marozaite, Egle Bareikiene, Vilius Rudaitis

**Affiliations:** ^1^ Faculty of Medicine Vilnius University Vilnius Lithuania; ^2^ State Research Institute Center for Innovative Medicine Vilnius Lithuania; ^3^ Clinic of Obstetrics and Gynecology, Faculty of Medicine, Institute of Clinical Medicine Vilnius University Vilnius Lithuania

**Keywords:** endometrial ablation, heavy menstrual bleeding, Lina Librata, minimally invasive surgery

## Abstract

**Objective:**

Heavy menstrual bleeding is one of the most common gynecologic disorders among women of reproductive age and has a significant impact on their physical, psychological, and social well‐being. Although international guidelines recommend medical therapy as the first‐line treatment, it may prove ineffective, poorly tolerated, or unsuitable due to comorbid conditions or concerns related to hormonal therapy. Minimally invasive, uterus‐preserving surgical interventions, such as endometrial balloon ablation, may represent an important therapeutic alternative; however, in clinical practice, its consideration and use remain limited. The aim of this study was to evaluate the effectiveness and safety of endometrial balloon ablation in a selected group of women with heavy menstrual bleeding.

**Methods:**

We conducted an observational study during the period 2022–2023, involving the collection and analysis of clinical data and patients' subjective evaluations of treatment outcomes at Vilnius University Hospital Santaros Klinikos. A total of 31 women presenting with heavy persistent menstrual bleeding were enrolled in the study and 28 women were included in final data analysis. All patients enrolled in our study had either experienced unsuccessful prior conservative medical treatment, had contraindications to first‐line treatment due to comorbidities, had personal reasons for declining hormonal treatment, or wished to preserve the uterus.

**Results:**

Endometrial ablation using the Lina Librata endometrial ablation system was performed in 28 patients. At 12 months of follow‐up, treatment success was achieved in 20 patients (71.43%; 95% confidence interval [CI]: 51.3–86.8), with procedure‐related amenorrhea in eight out of 20 patients (40%; 95% CI: 19.1–63.9). Treatment failure occurred in 28.57%; 95% CI: 13.2–48.6 (8/28) of patients; consequently, some patients declined further treatment for personal reasons, whereas others required or requested repeated ablative treatment or an alternative surgical intervention.

**Conclusion:**

Endometrial balloon ablation demonstrated a favorable safety and efficacy profile, and no complications occurred during the study period, including among patients considered to be at increased risk of bleeding. The procedure significantly contributes to uterus‐preserving treatment strategies and represents an appropriate alternative for women for whom medical therapy is ineffective or unsuitable, or who decline hysterectomy.

## 1 INTRODUCTION

Heavy, anemia‐inducing menstrual bleeding (menorrhagia) significantly impairs patients' physical, psychological, and social well‐being.^1^ According to previous study data, this condition affects up to one‐third of women of reproductive age. Women experiencing this disorder frequently report general weakness, reduced exercise tolerance, sudden fatigue, lack of energy, drowsiness, and other symptoms characteristic of anemia. These patients are often treated with long‐term iron supplementation, which is commonly associated with gastrointestinal adverse effects such as nausea, vomiting, and episodic abdominal pain.^2^ Heavy menstrual bleeding is a significant clinical concern and accounts for up to one‐third of gynecologic consultations annually.^3^ The most common treatments for heavy menstrual bleeding include a hormonal intrauterine device (IUD), combined oral contraceptives, extended‐cycle progestins, tranexamic acid, and nonsteroidal anti‐inflammatory drugs (NSAIDs). If such treatment proves ineffective, gonadotropin‐releasing hormone (GnRH) agonists or antagonists may be used. Third‐line treatment options include surgical interventions such as endometrial ablation, endometrial resection, and, ultimately, hysterectomy as a definitive therapeutic measure.^4^, ^5^ The management of heavy menstrual bleeding is often challenging, as currently available treatment options are not suitable or effective for all patients.^6^ Contemporary medicine emphasizes a patient‐centered approach that takes into account individual needs and personal preferences.^7^ Accordingly, the use of hormonal therapy for menorrhagia may be limited by patient concerns regarding potential adverse effects, the risk of hormone‐dependent cancers, or comorbid conditions that may preclude hormonal treatment (such as breast cancer, liver disease, or coagulative disorders).^8^, ^9^ Therefore, we chose to evaluate a uterus‐preserving, minimally traumatic, non‐hormonal treatment method – endometrial ablation.

The Lina Librata device is a single‐use medical instrument designed to thermally affect the endometrium. Following thermal treatment, the functional layer of the endometrium becomes significantly thinner, and in some cases, adhesions may form between the walls of the uterine cavity.^10^, ^11^ The procedure also affects the basal layer of the endometrium; consequently, the regenerating functional layer becomes substantially thinner, resulting in reduced menstrual bleeding, while in some patients, menstruation ceases entirely. Despite the well‐known processes of thermal injury to the endometrium, it is worth knowing that endometrial ablation does not always alleviate heavy bleeding during menstruation.^10^, ^12^, ^13^ Although this treatment modality is not new, it is infrequently recommended for various reasons, including relatively high cost and variability in access to healthcare services.^12^, ^13^, ^14^ This is reflected in the scarcity or outdated nature of both international and national publications on the subject.

The aim of our study was to evaluate the effectiveness of endometrial ablation as a non‐pharmacological treatment for menorrhagia.

## 2 MATERIALS AND METHODS

This prospective observational cohort study was conducted between 2022 and 2023 at the Center of Obstetrics and Gynecology of Vilnius University Hospital Santaros Klinikos, and involved the collection and analysis of clinical data and patients' subjective evaluations of treatment outcomes. The objective of the study was to evaluate whether endometrial ablation with a balloon system resulted in decreased menstrual bleeding. A total of 31 women presenting with heavy menstrual bleeding were enrolled in the study. The study included adult patients aged 18 years or older who had experienced unsuccessful conservative treatment or had contraindications to such therapy, had no desire for future pregnancy, and had no evidence of uterine or cervical malignancy. Endometrial ablation using the Lina Librata endometrial ablation system (CE 0459) was performed in 28 patients. Endometrial ablation is performed by introducing a thin balloon catheter through the cervix into the uterine cavity. Once the automatic safety checks are completed and the device is positioned, the balloon is filled with heated glycerine, enabling close contact with the endometrial surface. The generated heat produces controlled destruction of the endometrium, with the aim of reducing excessive menstrual bleeding. The procedure was carried out under intravenous anesthesia, following endometrial biopsy obtained during hysteroscopy. Treatment effectiveness was evaluated based on patient‐reported outcomes by answering yes/no questions, including whether menstrual bleeding had ceased or not and whether there was improvement in physical and psychological well‐being. In addition, procedure‐related pain was assessed using the visual analog scale (VAS). At 12 months, a dichotomous scale was used to assess patients' overall opinion of the procedure and its effectiveness.

Prior to the intervention, written informed consent was obtained from all participants for the treatment and for the use of their data in biomedical research. Approval for the study was granted by the Regional Biomedical Research Ethics Committee on November 22, 2022 (approval no. 2022/11‐1475‐946). Collected data were recorded, and statistical analysis was performed using Microsoft Excel and R Commander (version 2.9‐1).

## 3 RESULTS

A total of 28 patients were included in the final statistical analysis. The mean age was 45.46 years (range: 36–53 years). Prior to the procedure, endometrial biopsies were obtained hysteroscopically in all patients. Histologic examination revealed proliferative endometrium in 17 patients (60.71%), endometrial polyps in three (10.71%), secretory endometrium in two (7.14%), endometrial atrophy in four (14.29%), and leiomyoma in two (7.14%). The mean postoperative pain score was 2.89, assessed using the VAS. This finding indicates dull postoperative pain, generally lasting less than 24 h and effectively managed with oral NSAID therapy. Patient satisfaction was evaluated subjectively using a dichotomous scale (satisfied vs. dissatisfied). At 12 months, patients were contacted by telephone for follow‐up assessment. A significant reduction in menorrhagia or complete cessation of menstruation was achieved in 20 patients (71.43%; 95% confidence interval [CI]: 51.3–86.8) following endometrial ablation. In eight patients (28.57%; 95% CI: 13.2–48.6), the severity of heavy menstrual bleeding remained unchanged after the intervention. Among the 20 patients who achieved treatment success, complete absence of menstrual bleeding was observed in eight out of 20 patients (40%; 95% CI: 19.1–63.9). Three patients did not complete the study: one due to significant comorbidities (hypertrophic obstructive cardiomyopathy, myelodysplastic syndrome and myocardial infarction), one because endometrial balloon ablation could not be performed due to excessive uterine length (14 cm), as the automated Lina Librata system did not permit the intervention for safety reasons, and one patient did not present for the scheduled procedure and was therefore not included in the final analysis. During the monitoring period, four of the eight patients in whom the procedure was ineffective underwent repeated treatment due to persistent menorrhagia, while the remaining patients declined further intervention. This group of eight patients were classified as treatment failure in final statistical analysis. The full results and patient characteristics are presented in Table 1.

**TABLE 1:** Patient characteristics and results.

Patient characteristics	Variable + SD
Number of patients	28
Age	45.46 ± 4.54 (36–53)
Uterine length	8.80 ± 1.69 (6–13)
Parity	1.67 ± 0.72 (0–3)
Treatment before intervention	LNG‐IUS – 19 (67.86%) OCP – 9 (32.14%)
Histology	Proliferative endometrium 17 (60.71%) Endometrial polyp 3 (10.71%) Secretory endometrium 2 (7.14%) Leiomyoma 2 (7.14%) Endometrial atrophy 4 (14.29%)
Postoperative pain (VAS)	2.89 ± 1.85 (0–6)
Reduction in menstrual bleeding	20/28 patients (71.43%; 95% CI: 51.3–86.8)
Procedure‐related amenorrhea	8/20 patients (40%; 95% CI: 19.1–63.9)
Patient satisfaction in 12 months	20 satisfied (71.43%; 95% CI: 51.3–86.8) 8 dissatisfied (28.57%; 95% CI: 13.2–48.6)
Repeated treatment	3 hysterectomies 1 retreated with drospirenone
Complications	None
Comorbidities	History of thrombosis – 4 (14.29%) Chronic hepatitis C – 2 (7.14%) Liver cirrhosis – 2 (7.14%) Heart failure – 2 (7.14%)

Abbreviations: CI, confidence interval; LNG‐IUS, levonorgestrel releasing intrauterine system; OCP, oral contraceptive pills; SD, standard deviation; VAS, visual analog scale.

## 4 DISCUSSION

Heavy menstrual bleeding is one of the most common gynecologic disorders, affecting approximately 14%–30% of women of reproductive age.^15^ Management of this condition is multifaceted and guided by the recommendations of European obstetrics and gynecology societies and FIGO (International Federation of Gynecology and Obstetrics).^4^ In most cases, treatment is initiated with medical therapy. The first‐line treatment is a levonorgestrel‐releasing intrauterine device. Oral contraceptives, progestins, NSAIDs, and tranexamic acid are also widely used, while gonadotropin‐releasing hormone (GnRH) agonists or antagonists are generally reserved as a last‐line treatment option.^16^, ^17^ In cases of insufficient or ineffective medical treatment, uterus‐preserving minimally invasive surgical interventions may be considered, such as hysteroscopic endometrial resection or endometrial ablation using thermal, radiofrequency, or microwave energy to target the functional and basal layers of the endometrium, thereby reducing or eliminating menstrual bleeding.^5^, ^17^ Notably, systematic reviews indicate that endometrial ablation is considered a minimally invasive surgical treatment for heavy menstrual bleeding and is associated with low complication rates, mild postoperative pain, rapid recovery, and improved psychological and physical well‐being compared with those after a hysterectomy.^18^, ^19^

Based on this evidence, 31 patients were enrolled in our observational cohort study, of whom 28 were included in the final data analysis. The results of our study demonstrate that endometrial balloon ablation is a safe treatment option for a selected group of patients: women whose medical therapy was ineffective or poorly tolerated, those who declined hysterectomy, and those for whom major surgical intervention, such as hysterectomy, posed a significant risk to life. It is worth noting that some patients were receiving anticoagulant therapy due to prior or existing comorbidities; nevertheless, no procedure‐related complications were observed, and the treatment remained effective. Compared to a study by Guyer, which evaluated 52 patients treated using the same ablation system, where patient satisfaction reached 81%, and amenorrhea was achieved in 33% of cases.^20^ In our study, overall satisfaction was lower (71.43%); however, the rate of procedure‐related amenorrhea was higher (40% vs. 33%). This difference may be explained by the longer follow‐up period in our study (12 months vs. 6–9 months), as endometrial regeneration and the final therapeutic effect may become apparent over time.^21^ No complications were reported in either study. Clinical data suggest that endometrial ablation devices have a favorable safety profile. Due to integrated safety mechanisms, the device continuously monitors procedural parameters in real time and automatically determines whether conditions are appropriate to safely perform ablation.^22^ Despite minor differences between the studies, the overall trends remain comparable, supporting the conclusion that endometrial balloon ablation is a reliable and effective treatment method.

Similar findings were reported by Fortin in a prospective study involving 20 women with severe menstrual bleeding. The study evaluated the effectiveness of endometrial ablation performed in an outpatient setting under local anesthesia or sedation. After 6 months of follow‐up, bleeding decreased in all patients, and amenorrhea was achieved in 30% of cases (6/20). All participants reported high satisfaction with the procedure (89.5% based on subjective assessment).^23^ In our observational study, treatment effectiveness at 12 months was observed in 71.43% of patients, representing the lowest rate among the published comparative studies.^20^, ^23^ This difference is likely attributable to the stricter evaluation methodology applied in our study and enrolment of patients with history of chronic hepatitis C, liver cirrhosis, heart failure and past thrombotic events. It is worth knowing that in the study by Fortin the menstrual pictogram (MP) and menorrhagia multi‐attribute scale (MMAS) was used at baseline and after 6 months to determine the effectiveness of the procedure. It is also important to point out that the data in the studies by Fortin and Guyer were presented in a conference format rather than a peer‐reviewed publication, and these results should be interpreted as supportive same‐device clinical experience rather than as a direct methodological comparator.

A new approach was chosen in our study to evaluate treatment effectiveness which was assessed using a predefined dichotomous scale based on clearly defined clinical and patient‐reported outcomes, rather than broader subjective interpretations. Many studies evaluating the effectiveness of endometrial ablation include bleeding patterns (volume, quantity, regularity, etc.), which may lead to higher reported treatment success if any of these parameters improve. In our study, effectiveness was assessed differently. The evaluation was limited to the presence or absence of menstrual bleeding; furthermore, the procedure's impact on daily and psychosocial functioning, based on patients' explicit assessment, was included. We believe that this standardized evaluation model reduces interpretative variability and may more accurately reflect procedural effectiveness in real‐world clinical practice.

Our study also included patients from higher‐risk groups receiving anticoagulant therapy due to coagulation disorders or comorbid conditions; such groups were not described in the studies by Fortin or Guyer. In addition, our procedures were performed in the operating room under intravenous anesthesia, which may have influenced postoperative pain assessment. Despite these differences, no complications were reported in either study. Postoperative pain was minimal (mean VAS score < 3), and the results consistently demonstrate the safety and effectiveness of the Lina Librata system in the treatment of heavy menstrual bleeding, particularly as an alternative to hysterectomy in higher‐risk populations. An important issue for discussion is the ability to conceive after endometrial ablation. The median age of our study patients was 45 years old, although a small number were 36 years old. Counseling regarding future reproductive plans is essential and is often challenging because of limited scientific evidence evaluating the rates of pregnancies and its outcomes after endometrial ablation. A systematic review was conducted by Kohn et al. which identified that 85% of pregnancies described in observational studies after endometrial ablation had ended in termination, miscarriage or ectopic pregnancies. Among the ones that continued, high rates of preterm delivery, cesarean delivery, cesarean hysterectomy or morbidly adherent placenta were observed.^24^ Therefore, we discussed this issue with our patients and recommended declining ablative treatment for those who wished to preserve the ability to conceive. Thus, it is important to inform patients that ablative treatment does not provide contraception; therefore, additional methods should be considered.

### 4.1 Strengths and limitations

A major strength of our study is its focus on real‐life situations encountered in clinical practice. All patients presenting with heavy menstrual bleeding were included, without exclusion based on existing or comorbid conditions that could potentially influence treatment outcomes. This patient population represents a significant clinical challenge in everyday practice; therefore, our findings have considerable practical relevance. The study was methodologically consistent, as the intervention was performed using the same Lina Librata system in all patients.

However, the study had major limitations. First, the number of patients included was relatively small, which limits statistical power. Second, patients were not divided into comparison groups, which restricts the ability to compare endometrial ablation procedure with other treatment modalities. Third, the study did not include objective criteria for treatment success, such as the pictorial blood assessment chart or menstrual pictogram score. This represents a major methodological limitation, as the use of a validated bleeding assessment tool could have provided a more standardized and quantifiable evaluation of treatment outcomes.

However, we would like to address that the primary aim of the study was to assess treatment effectiveness from the patient's own perspective, focusing on whether the procedure improved bleeding symptoms sufficiently to positively affect daily life and overall well‐being. Heavy menstrual bleeding is a condition in which the perceived burden of symptoms and their impact on daily activities are different patient to patient and are highly relevant to treatment satisfaction. Therefore, we chose to rely on patients' subjective assessment of improvement rather than requiring additional structured recording of menstrual blood loss.

In addition, this was a single‐center study with a heterogeneous patient population.

## 5 CONCLUSION

Endometrial balloon ablation using the Lina Librata system is a safe and effective minimally invasive alternative for the treatment of heavy menstrual bleeding. In our study, treatment effectiveness reached 71.43% (20/28), 95% CI: 51.3–86.8, with procedure induced amenorrhea of 40% (8/20), 95% CI: 19.1–63.9, after 12 months and no observed complications, including among patients with an increased risk of bleeding. The procedure is particularly suitable for women for whom medical therapy is ineffective or who decline hysterectomy. These results support the clinical value of endometrial ablation in selected patients. Our findings highlight the importance of discussing realistic procedural outcomes with patients, as high expectations may not always be fulfilled owing to the various underlying factors contributing to the etiology of menorrhagia. Larger prospective studies are recommended to further evaluate its long‐term outcomes.

## AUTHOR CONTRIBUTIONS

The original article idea was proposed by VR and EB. Planning and conducting this observational study was done by VR and EB. Data collection was done by VR. Anonymized data analysis was done by JR and PM. Manuscript was written by JR and PM supervised by VR and EB. All authors were involved in editing the document and approved the final version before publication.

## FUNDING INFORMATION

This research received no external funding.

## CONFLICT OF INTEREST STATEMENT

The authors declare that they have no conflicts of interest.

## Data Availability

Data sharing is applicable if necessary.
